# Assortative mating by colored ornaments in blue tits: space and time matter

**DOI:** 10.1002/ece3.2822

**Published:** 2017-02-26

**Authors:** Amélie Fargevieille, Arnaud Grégoire, Anne Charmantier, Maria del Rey Granado, Claire Doutrelant

**Affiliations:** ^1^ CEFE UMR5175CNRS – Université de MontpellierMontpellierFrance

**Keywords:** color traits, *Cyanistes caeruleus*, mate choice, pairing association, secondary sexual characters

## Abstract

Assortative mating is a potential outcome of sexual selection, and estimating its level is important to better understand local adaptation and underlying trait evolution. However, assortative mating studies frequently base their conclusions on small numbers of individuals sampled over short periods of time and limited spatial scales even though spatiotemporal variation is common. Here, we characterized assortative mating patterns over 10 years in four populations of the blue tit (*Cyanistes caeruleus*), a passerine bird. We focused on two plumage ornaments—the blue crown and the yellow breast patch. Based on data for 1,657 pairs of birds, we found large interannual variation: assortative mating varied from positive to negative. To determine whether there was nonetheless a general trend in the data, we ran a within‐study meta‐analysis. It revealed that assortative mating was moderately positive for both ornaments. It also showed that mating patterns differed among populations and especially between two neighboring populations that displayed phenotypic divergence. Our results therefore underscore that long‐term studies are needed to draw broad conclusions about mating patterns in natural populations. They also call for studying the potential role of assortative mating in local adaptation and evolution of ornaments in both sexes.

## 1 Introduction

Studying sexual selection in the wild poses several methodological challenges. In particular, in animal species with separate sexes, measuring the direction and force of intra‐ and intersexual selection requires collecting information on difficult‐to‐quantify behaviors from large numbers of individuals. An alternative approach is to study the outcome of selection via mating patterns and more specifically positive or negative assortative mating, which is when an organism pairs with a similar or dissimilar mate, respectively (Jiang, Bolnick, & Kirkpatrick, 2013).

Assortative mating may be the consequence of many factors such as habitat choice or timing of breeding but is often a consequence of mate choice (Andersson, 1994; Price, 2008). It is relatively straightforward to study assortative mating, and it has been the subject of much short‐term research conducted at small spatial scales (Jiang et al., 2013). However, the conclusions of such work may be unreliable because key studies conducted on traits subject to sexual selection have clearly shown that mate choice can vary in space and time (Svensson & Gosden, 2007). For instance, female preferences for male body size vary greatly among neighboring populations of the damselfly *Ischnura elegans* (Svensson & Gosden, 2007). Likewise, severe drought can cause atypical dispersal events that fragment group structure and thus affect the direction of sexual and kin selection in the white‐winged chough, *Corcorax melanorhampos*, a cooperative breeder (Heinsohn, Dunn, Legge, & Double, 2000). Because most studies examining secondary sexual traits in general, and assortative mating in particular, are carried out at a small number of sites and over very few breeding seasons (Cornwallis & Uller, 2010 but see Cockburn, Osmond, & Double, 2008; Svensson, Abbott, Härdling, Losos, & Moore, 2005), longer‐term research that spans broader spatial scales is needed if we wish to more accurately estimate the strength and consequences of mating patterns.

Assortative mating has broad‐ranging consequences on trait evolution, local adaptation, and speciation. Theoretical models have shown that, when it is associated with disruptive selection, assortative mating by single traits or multiple ecologically related traits facilitates sympatric speciation (Bolnick & Fitzpatrick, 2007; Dieckmann & Doebeli, 1999; Gavrilets, 2003) and local adaptation (Jiang et al., 2013). In a *Tilapia* species complex in the early stages of speciation, there was strong assortative mating by diet and color, which may promote species differentiation (Martin, 2013). However, in general, there is limited empirical evidence for assortative mating playing such a role (Branch, Kozlovsky, & Pravosudov, 2015; Nosil, Egan, & Funk, 2008). One reason may be the existence of large temporal variation in assortative mating patterns: studies conducted over 1 or 2 years are unlikely to be able to provide a representative view of long‐term patterns (Cockburn, 2014). To date, temporal variation in assortative mating patterns has remained unaddressed.

Assortative mating also influences the evolution of secondary sexual characters in both sexes and therefore must be characterized if we wish to understand the basis for female ornamentation. Indeed, quantifying mating patterns is the first step in disentangling the still‐debated factors underlying the evolution of female ornaments. For instance, female ornaments may be an evolutionary by‐product resulting from selection for male ornaments and/or emerge from direct sexual or social selection on female traits (Clutton‐Brock, 2007; Tobias, Montgomerie, & Lyon, 2012).

In animals, both sexes often convey multiple signals to conspecifics; these signals may provide redundant or complementary information about an individual's quality. Therefore, males and females may assess each other using different signals and/or different aspects of the same signal. However, few studies have investigated whether the two sexes differ in the signals/signal aspects they use (Hegyi et al., 2015; Laczi, Török, Rosivall, & Hegyi, 2011). When studying coloration, the influence of different signals on mate choice is difficult to unravel because of multicollinearity among traits. In this study, we used a commonality analysis to identify potential multicollinearity among explanatory variables (Ray‐Mukherjee et al., 2014). Exploring trait associations can help reveal any complexity hidden in mating patterns.

Evidence for assortative mating is usually based on the value of the Pearson correlation coefficient for pairs of traits. Although it is straightforward to calculate, its use may be problematic when dealing with longitudinal data because of the risk of pseudoreplication. The most common solution is to use the trait value for a given individual for 1 year only; frequently, researchers choose the value associated with the individual's first appearance or with a randomly selected time period (Row & Weatherhead, 2011; Van Rooij & Griffith, 2012). Although this approach prevents pseudoreplication, it dramatically decreases sample size. Furthermore, if an individual changes mates every breeding season, the strategy leads to a major loss of information, making it harder to accurately estimate the degree of interannual variability. To assess the strength of assortative mating, we employed a powerful but rarely applied method that can be used with multiple populations of a single species: the within‐study meta‐analysis. This approach allowed us to draw generalized conclusions while avoiding pseudoreplication (Nakagawa & Santos, 2012).

**Figure 1:**
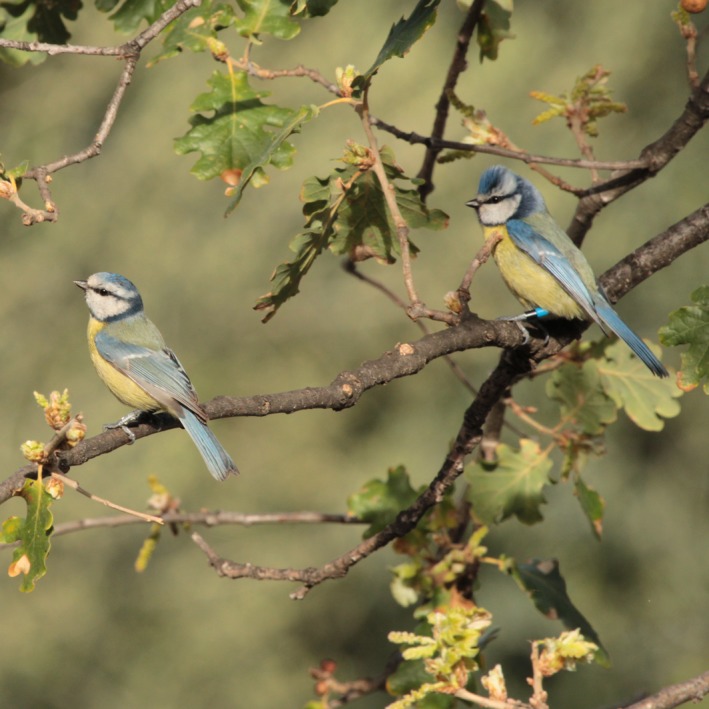
Pair of blue tits (female on the left, male on the right) photographed in the Regino valley (around Muro field stations) in Corsica in March 2016. Picture courtesy of Stéphan Tillo

We studied assortative mating in the blue tit (*Cyanistes caeruleus*), a passerine. Mate choice is likely important in this socially monogamous species, in which birds pair before the breeding season and both sexes provide parental care (see Figure 1). We focused on two ornaments—the blue/UV crown, which is produced by structural coloration, and the yellow breast patch, which results from carotenoid‐based coloration. Both features are thought to be targets of social or sexual selection in both males and females (Alonso‐Alvarez, Doutrelant, & Sorci, 2004; Limbourg, Mateman, Andersson, & Lessells, 2004; Kingma et al., 2009; Remy, Gregoire, Perret, & Doutrelant, 2010 but see Parker, 2013 for males; Doutrelant, Grégoire, Midamegbe, Lambrechts, & Perret, 2012; Doutrelant et al., 2008; Henderson, Heidinger, Evans, & Arnold, 2013; Midamegbe, Grégoire, Perret, & Doutrelant, 2011; Midamegbe et al., 2013; Parker et al., 2011 for females). If ornaments influence mate choice differently for males and females, multicollinearity can occur among traits. Consequently, we anticipated that the final pattern might be more complex than that normally expected from “simple” assortative mating.

Two previous studies on assortative mating in blue tits (Andersson, Örnborg, & Andersson, 1998; Garcia‐Navas, Ortego, & Jose Sanz, 2009) focused on a single ornament, the blue crown, and obtained data from a single population (using 18 and 26 breeding pairs, respectively) over a single breeding season. They found opposite results: Andersson et al. (1998) found evidence for positive assortative mating, whereas Garcia‐Navas and colleagues did not. Here, not only did we use two ornaments, but we also collected data from four different populations over 10 successive breeding seasons; we sampled a mean of 44 breeding pairs per year per population. Long‐term studies that compile large data sets are essential if we wish to better understand the potential strength of social and sexual selection on ornaments (Parker, 2013). Also, as assortative mating is a force that contributes to local adaptation (Jiang et al., 2013), it is crucial to assess the former's strength in closed populations that display phenotypic divergence. Of our four study populations, two were separated by less than 5 km and experienced gene flow (Szulkin, Gagnaire, Bierne, & Charmantier, 2016); however, they nonetheless diverged in several fitness‐related life‐history traits, including coloration (Charmantier, Doutrelant, Dubuc‐Messier, Fargevieille, & Szulkin, 2016). They thus present an ideal opportunity for investigating the link between assortative mating and local adaptation.

## 2 Materials and Methods

### 2.1 Study populations and sampling methodology

From 2005 to 2014, we collected feathers from birds in four blue tit populations in France. One was located in mainland France, in a deciduous oak forest (La Rouvière) found approximately 18 km northwest of Montpellier. The other three were located in northwestern Corsica. Of the Corsican populations, two were found in the Regino Valley (the Muro populations) and were separated by less than 5 km. Despite the populations' spatial proximity, they occupied different habitat types: a deciduous oak forest versus an evergreen oak forest (Blondel et al., 2006; Charmantier, Doutrelant, et al., 2016; Lambrechts et al., 2004). The third Corsican population, the Pirio population, was found in the Fango Valley, which is located 25 km from the Regino Valley, in a forest dominated by evergreen oaks. Hereafter, the four populations are referred to as D‐Rouvière, D‐Muro, E‐Muro, and E‐Pirio, where D stands for deciduous oaks and E stands for evergreen oaks.

We sampled feathers from breeding pairs that were trapped in their nestboxes as they fed their chicks, approximately 9 days after the chicks hatched. Sampling took place in late April and May for the D‐Rouvière, D‐Muro, and E‐Muro populations and in June for the E‐Pirio population. Because feather coloration changes over time as feathers become soiled (Delhey, Peters, Johnsen, & Kempenaers, 2006; Ornborg, Andersson, Griffith, & Sheldon, 2002), different mating patterns could be present at the beginning versus the end of the breeding season. Feathers were collected from a small number of breeding pairs both during the nest construction period and the chick feeding period of the same season to determine whether there was evidence for intraseasonal differences in assortative mating patterns and strength among the study populations. Although assortative mating increased in strength within breeding seasons, the population‐level patterns were the same as in the more extensive analysis, suggesting sampling period was not a concern (see Appendix S1 for more details on the methods and results).

Upon capture, each bird was equipped with a uniquely numbered metal band provided by the French Natural History Museum in Paris; this effort was part of the CRBPO banding program (#369). We collected 8 and 10 feathers from each bird's blue crown and yellow breast patch, respectively (Doutrelant et al., 2008, 2012).

### 2.2 Measurements of color traits

We characterized five color traits that have been examined in previous studies of blue tit populations (Alonso‐Alvarez et al., 2004; Delhey, Johnsen, Peters, Andersson, & Kempenaers, 2003; Doutrelant et al., 2008, 2012; Limbourg et al., 2004; Midamegbe et al., 2011, 2013; Remy et al., 2010; Sheldon, Andersson, Griffith, Örnborg, & Sendecka, 1999). Measurements were performed in the laboratory using a spectrophotometer (AVASPEC‐2048; Avantes, Apeldoorn, the Netherlands) and a deuterium–halogen light source (AVALIGHT‐DH‐S lamp; range of 300–700 nm; Avantes, Apeldoorn, the Netherlands). A 200‐μm fiber optic probe was placed perpendicular to the feather's surface at a fixed distance of 2 mm. A probe mount consisting of a black rubber cap excluded all ambient light. Our reflectance data (R) were relative to a white standard (WS‐1; Ocean Optics, Dunedin, FL, USA) and a dark current (a black felt background). We measured each sample three times, the fiber being removed between each measure. For each ornament on each bird, we made measurements on two different samples and found the mean of six reflectance spectra obtained from two sets of three blue feathers and four yellow feathers (Doutrelant et al., 2008, 2012). Samples within a year and a population were measured randomly, controlling a potential effect of random drift due to measuring male and female samples consecutively. We used Avicol v6 software (Gomez, 2006) to estimate the color trait values based on the shape of the spectra (Andersson et al., 1998; Doutrelant et al., 2008).

In the case of the blue crown, we characterized one achromatic trait, blue brightness (i.e., the area under the reflectance curve divided by the width of the interval from 300–700 nm), and two chromatic traits, blue hue (wavelength at maximal reflectance) and blue UV‐chroma (proportion of the total reflectance falling in range from 300–400 nm). Lower hue values and higher UV‐chroma values correspond to a stronger UV signal. In the case of the yellow breast patch, we calculated yellow brightness and yellow chroma ([*R*_700_ − *R*_450_]/*R*_700_). Higher yellow chroma values are associated with higher levels of carotenoids in the plumage (Isaksson, Ornborg, Prager, & Andersson, 2008). Our measurements were all highly repeatable, suggesting acceptable measurement error (Appendix S2, Table S2.1).

### 2.3 Assortative mating database characteristics

In all our analyses, we only used pairs for which coloration data was available for both the male and the female. Most of the time (97.4%), feathers were collected from both members of a mated pair within 3 days. Within each population and breeding season, pairs that reproduced 30 days after their first attempt were excluded from the analysis because such clutches were potentially second or replacement clutches (Nager & Vannoordwijk, 1995).

Between 2005 and 2014, we analyzed color trait values for 1,657 mated pairs (Table 1), including 1,117 males and 1,110 females. The correlation (Pearson correlation coefficient) between trait values never exceeded 0.42 (Appendix S2, Table S2.2), except in the case of blue hue and blue UVchroma, which ranged from −0.33 to 0.74 (Appendix S2, Table S2.2).

**Table 1:** Sample sizes for each population and year; total sample sizes are in bold

	D‐Muro	E‐Muro	D‐Rouvière	E‐Pirio
2005	33	19	62	46
2006	39	24	47	37
2007	0	0	41	58
2008	39	23	51	33
2009	37	26	42	37
2010	54	30	65	50
2011	55	32	76	51
2012	58	31	56	29
2013	50	30	44	38
2014	54	39	84	37
Total	**419**	**254**	**568**	**416**

### 2.4 Statistics

#### 2.4.1 Commonality analysis

We used commonality analysis to explore trait associations (i.e., which predictors explain most of the variance in a given dependent variable) and to identify potential multicollinearity (Ray‐Mukherjee et al., 2014). We used the five standardized color traits for one sex as predictors in additive multiple regression models in which the trait values for the other sex were dependent variables (Table 2). The commonality analysis was then performed on each of the 10 multiple regression models using the *yhat* package (Nimon, Oswald, & Roberts, 2013) in R (R Core Team 2016) to determine which variables should be retained in the analyses described below.

**Table 2:** Percentage of variance explained by the concordant color trait in the opposite sex

Dependent variable	Predictor variables in the additive model	Concordant color trait	Variance explained (%)
Female blue brightness (fBB)	mBB + mBH + mBUVC + mYB + mYC	mBB	72
Female blue hue (fBH)		mBH	75
Female blue UV‐chroma (fBUVC)		mBUVC	78
Female yellow brightness (fYB)		mYB	89
Female yellow chroma (fYC)		mYC	92
Male blue brightness (mBB)	fBB + fBH + fBUVC + fYB + fYC	fBB	82
Male blue hue (mBH)		fBH	75
Male blue UV‐chroma (mBUVC)		fBUVC	92
Male yellow brightness (mYB)		fYB	67
Male yellow chroma (mYC)		fYC	86

#### 2.4.2 Characterizing assortative mating patterns

To look for evidence of assortative mating, for each population and each year, we calculated the Pearson correlation coefficients between the trait values for mated pairs; only the traits retained following the commonality analysis were used. As we were working with as many as five color traits for birds from four populations sampled across 10 years, a within‐study meta‐analysis was best suited for detecting potential patterns of assortative mating (Hegyi et al., 2015; Nakagawa & Santos, 2012). This method takes into account any nonindependence in the data within populations and among variables. The Pearson correlation coefficients and the confidence intervals we had calculated were transformed to fit the meta‐analysis and were then used as effect sizes (Rosenberg, Rothstein, & Gurevitch, 2013). We included year as a random effect.

In the first set of models, color trait and population were fixed effects; their interaction was also included. Then, ornament variation was examined by grouping the blue crown traits and yellow breast patch traits to create two levels of a new variable: ornament. Ornament and population were then included as fixed effects, as was their interaction. Models were run using the *MCMCglmm* package (Hadfield, 2010) in R; 100,000 iterations were performed, with a burn‐in of 25,000 iterations. We used a DIC model selection approach. The results of the best‐fit models were back‐transformed to obtain new Pearson correlation coefficients (Nakagawa & Santos, 2012; Rosenberg et al., 2013).

We examined temporal variation by two means. We first tested for temporal autocorrelation among Pearson correlation coefficients on each color trait in each population. For the D‐Muro and E‐Muro populations, due to the lack of data in 2007, we estimated temporal autocorrelation from 2008 to 2014. We also used a within‐study meta‐analysis model that included year as a fixed effect rather than as a random effect, which permitted the inclusion of year‐by‐population and year‐by‐ornament interactions.

## 3 Results

### 3.1 Commonality analysis

For each of the 10 multiple regression models tested in the commonality analysis, a given color trait in one sex always explained most of the variance in the concordant trait in the other sex. Explanatory ability ranged from 67% for yellow brightness to 92% for blue UV‐chroma and yellow chroma (Table 2; see Appendix S2, Table S2.3 for details). This result means that corresponding traits (e.g., male blue brightness and female blue brightness) were more likely to be associated than were noncorresponding traits (e.g., male blue brightness and female blue UV‐chroma). As a result, we only retained concordant traits in our analyses of assortative mating patterns.

### 3.2 Pearson correlation coefficients

**Figure 2:**
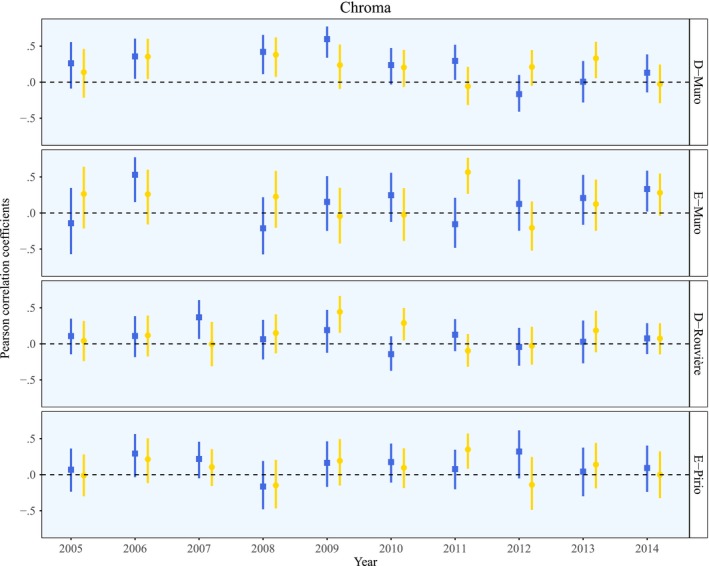
Pearson correlation coefficients for blue UV‐chroma (blue squares) and yellow chroma (yellow circles) for each year for each population. The bars depict the 95% confidence intervals. The dashed lines indicate a coefficient value of zero, or the absence of assortative mating

For each population and each trait, there was notable variation among years in the strength of assortative mating (see Figure 2 for blue UV‐chroma and yellow chroma; see Appendix S2, Fig. S2.1 for the other three traits). The most positive values were around 0.60 (i.e., 0.60 for blue UV‐chroma in D‐Muro in 2009 and 0.57 for yellow chroma in E‐Muro in 2011), and the most negative values were around −0.40 (−0.39 for blue brightness in E‐Pirio in 2008).

### 3.3 Temporal autocorrelation

Overall, there was no effect of temporal autocorrelation among years for every population and every color trait. Only a marginal negative effect of temporal autocorrelation for yellow brightness was found in E‐Pirio with a lag of 2 years (see Fig. S2.2).

### 3.4 Within‐study meta‐analysis

**Figure 3:**
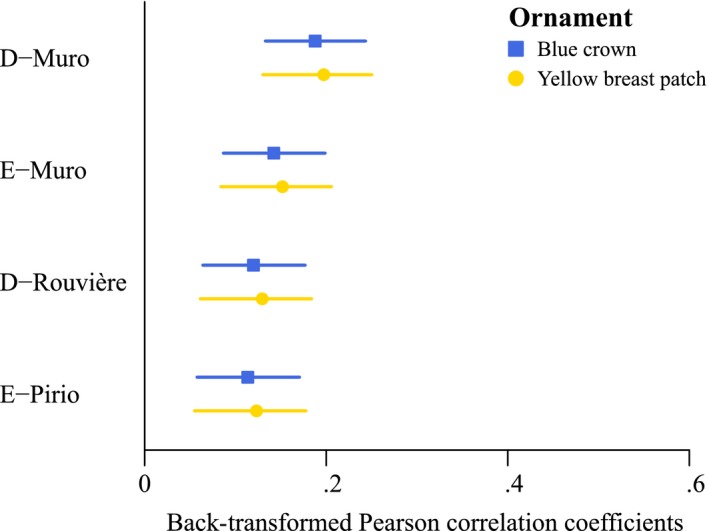
Back‐transformed population‐specific Pearson correlation coefficients for the two ornaments: the blue crown (blue squares) and the yellow breast patch (yellow circles). The bars depict the 95% confidence intervals

The within‐study meta‐analysis showed that, on the whole, assortative mating was positive in all four populations, even though there was a large degree of temporal variation. In the analysis in which the color traits were included separately, the best‐fit model included only population (Table 3a), which means that there was variation in assortative mating among populations but not among color traits (Figure 2 and Appendix S2, S2.1). More specifically, assortative mating was strongest in the D‐Muro population and weakest in the D‐Rouvière and E‐Pirio populations. All the populations had assortative mating values of 0.2 or less (Figure 3), but the values were all significantly different from zero, indicating that the two ornaments were serving as the basis for assortative mating.

**Table 3:** Meta‐analysis models in which year was a random effect

Fixed effects	Random effect	DIC
a) Traits included separately		
Population	Year	**−478.8**
Trait + population	Year	−455.1
Intercept	Year	−454.2
Trait	Year	−441.2
Trait × population	Year	−404.0
b) Traits grouped by ornament		
Ornament + population	Year	−**468.9**
Population	Year	−460.5
Ornament	Year	−448.1
Ornament × population	Year	−447.6
Intercept	Year	−445.7

The best‐fit model (in bold) was the model with the lowest DIC value. Ornament had two levels: blue crown and yellow breast patch.

In the analysis in which the color traits were grouped, the best‐fit model included ornament and population as main effects (Table 3b). Looking more closely at the results, there appeared to be somewhat stronger assortative mating by the yellow breast patch than by the blue crown (Figure 3).

In the analysis in which year was treated as a fixed effect, the best‐fit model included ornament and population as main effects but did not retain their interaction, which suggests temporal variation was similar across all the four populations (Appendix S2, Table S2.4 and Fig S2.3).

## 4 Discussion

This study investigated spatiotemporal variability in assortative mating in four blue tit populations in southern France; it focused on two ornaments, the blue crown and the yellow breast patch. We found four major results. First, concordant color traits were highly associated in breeding pairs; for instance, males with bright blue crowns paired with females with bright blue crowns. Second, on the whole, assortative mating was positive in all four populations, suggesting it influences ornament evolution in both sexes in this species. Assortative mating was slightly more pronounced for the yellow breast patch than for the blue crown. Third, we observed large temporal variation in assortative mating patterns in the four populations, a result that underscores that long‐term studies are needed to draw conclusions about mate pairing in natural populations. Fourth, there was spatial variation in assortative mating strength. The two closest populations displayed the highest levels of assortative mating, which may have interesting evolutionary implication on local adaptation and population divergence in our study system.

### 4.1 Assortative mating is based on concordant color traits

First, our results show that both sexes use the same color traits to choose mates. In the commonality analysis, concordant color traits in males and females were always much more strongly associated than were nonconcordant color traits. For instance, when all five female color traits were used as predictors of male yellow chroma, female yellow chroma alone explained 86% of the variation (Table 2 and Appendix S2, Table S2.3). This lack of multicollinearity also suggests that different traits do not form a general, integrated signal. This finding is in line with recent discoveries in great tits and collared flycatchers (Hegyi et al., 2015; Laczi et al., 2011).

Second, in all populations, there was a slightly greater degree of assortative mating by the yellow breast patch than by the blue crown. This result could suggest that both ornaments convey different information and that the yellow breast patch might be more important in blue tit social and/or sexual interactions. Alternatively, if assortative mating is related to sharing the same habitat (Kraaijeveld, Kraaijeveld‐Smit, & Komdeur, 2007), a stronger assortative mating by the yellow breast patch could mean that the yellow breast patch is more condition‐dependent than the blue crown (due to its sensitivity to food availability—carotenoid—and parasites). Yet even if the best‐fit meta‐analysis model retained a population effect (Table 3b), the ornament‐specific differences in assortative mating values were small and their confidence intervals overlapped (Figure 3). Moreover, when the five color traits were treated as individual factors, the best‐fit meta‐analysis model did not retain any of them (Table 3a). More studies are thus needed to determine the role of sharing the same habitat on assortative mating value and to determine whether the two ornaments convey different or similar information. More specifically, it would be useful to carry experimental studies in which the ornaments are manipulated (Jawor & Breitwisch, 2004) to answer this question.

### 4.2 Assortative mating is positive overall

We found that, on the whole, assortative mating by the blue crown and by the yellow breast patch was positive in the four study populations. Although its strength never exceeded 0.2 across the 10 years of the study, it was nonetheless significantly different from zero in each population. The large temporal variation observed may help explain why its mean values were relatively low, even though it reached a maximum of 0.6 (Figure 2 and Appendix S2, Fig. S2.1).

This overall level of assortative mating is slightly lower than what has been found for birds in general, and for bird visual signals in particular, in a recent meta‐analysis of results from natural populations (*r* =0.25 with [0.20;0.29] 95% confidence intervals and *n* = 132 for all traits in birds; *r* =0.37 with [0.26;0.48] 95% confidence intervals and *n* = 14 for visual signals in birds; see ESM B tables B1 and B4 in Jiang et al., 2013). However, most studies of color signals in birds last no more than three years (but see Roulin, Ducret, Ravussin, & Altwegg, 2003; Brommer, Ahola, & Karstinen, 2005), and because there is a strong publication bias for significant results in science (Palmer, 1999), these effects are probably overestimates.

In the context of female ornament evolution, positive assortative mating by ornaments could be strongly related to sexual selection (e.g., the mate choice hypothesis) or social selection (e.g., the competition hypothesis; Amundsen, 2000; Clutton‐Brock, 2007, 2009; Dale, Dey, Delhey, Kempenaers, & Valcu, 2015; Tobias et al., 2012). If assortative mating is a consequence of mate choice (either directional choice or mutual mate choice), then choosing a mate based on the similarity of his/her ornaments ultimately favors the presence of the ornament in the mate. Likewise, if assortative mating is a consequence of competition (either for territory or resources), trait similarity could result because the same signal conveys information about competitive status in both males and females. Previous results obtained in the blue tit have suggested that female coloration on the blue crown plays a role in male mate choice and competition (Alonso‐Alvarez et al., 2004; Hunt, Cuthill, Bennett, & Griffiths, 1999; Midamegbe et al., 2011; Remy et al., 2010). It has also been shown experimentally that female coloration on the yellow breast patch is condition‐dependent and related to reproductive success and maternal reproductive investment (Doutrelant et al., 2008; Midamegbe et al., 2013). Our results thus join with others to suggest that female ornaments in this species may be directly selected. Of course, this does not preclude genetic correlations from playing a role in the evolution of female ornaments, especially because strong additive genetic correlations in chromatic traits have been found between the sexes in our study populations (Charmantier, Wolak, Grégoire, Fargevieille, & Doutrelant, 2017).

### 4.3 Large temporal variation exists in assortative mating

A longitudinal study examining multiple ornaments in a population of lark buntings (*Calamospiza melanocorys*) found that sexual selection on male traits shifted dramatically over time and even demonstrated reversals in directionality for given traits (Chaine & Lyon, 2008). Similarly in our study, the raw Pearson correlation coefficients for the individual populations revealed large temporal variation in the strength of assortative mating by color traits. For instance, in the D‐Muro population in 2011, there was significant positive assortative mating by blue UV‐chroma (*r*_BUVC_ =0.29; Figure 2) but not by yellow chroma (*r*_YC_ =0−.06; Figure 2). In the same population in 2013, the opposite was observed (*r*_BUVC_ =0.006; *r*_YC_ =0.33; Figure 2). This finding illustrates that we should not draw general conclusions about assortative mating based on a single breeding season because results may vary substantially from year to year. In the review written by Jiang et al. (2013) on the topic of assortative mating, most of the studies cited lasted no longer than a year.

Although 10 years' worth of variation is insufficient if we wish to draw conclusions about the potential ecological factors driving variability, the results of the analysis in which year was treated as a fixed effect suggest that fluctuations in assortative mating follow similar patterns across the four study populations (Appendix S2, Fig S2.3) and could therefore be explained by common ecological factors. This similar year effect suggests that the large temporal variation observed is more than just random fluctuation around the mean; furthermore, the lack of temporal autocorrelation (Fig. S2.2) indicates that patterns of assortative mating were independent across years.

To explain such interannual variability, we speculate that the dramatic variation in the strength of assortative mating by the two ornaments and their color traits could be influenced by interannual plasticity in mate choice (Chaine & Lyon, 2008; Kopp & Hermisson, 2008). This plasticity could stem from the fact that although the two ornaments are condition‐dependent (in adults: Doutrelant et al., 2012 but see Peters, Kurvers, Roberts, & Delhey, 2011; in nestlings: Jacot & Kempenaers, 2007), they display differential abilities to respond to environmental conditions and are thus more or less informative depending on the main factors affecting signaling in any given year (Bro‐Jørgensen, 2010). For instance, variable carotenoid availability or parasite prevalence during the molt may mainly affect the reliability of the yellow breast patch as a signal while leaving less mark on the blue crown, whose coloration is structural (McGraw, Mackillop, Dale, & Hauber, 2002). Variation in mortality and density may also affect mate choice or competitive intensity, leading to a lesser degree of assortative mating during years when conditions are harsher (Crowley et al., 1991).

### 4.4 Large spatial variation exists in assortative mating

Theory predicts that local adaptation will be reinforced when assortative mating occurs (Jiang et al., 2013). Interestingly, we found a greater degree of assortative mating in the two populations closest to each other: the Corsican Muro populations. They display hints of local adaptation and strong phenotypic divergence for several morphological, behavioral, and life‐history (i.e., laying date, clutch size, fledgling success) traits (Charmantier, Doutrelant, et al., 2016; Porlier, Garant, Perret, & Charmantier, 2012; Szulkin et al., 2016).

The Muro populations are separated by no more than 5 km and, although dispersal between the two has been observed, recent genomic studies have revealed that the populations display fine‐scale genetic structures (Szulkin et al., 2016), which suggests the presence of forces limiting random dispersal. The positive assortative mating we observed in these populations may be one of those forces. These results, taken in tandem with those of a previous study that found population‐level differences in coloration (Charmantier, Doutrelant, et al., 2016), indicate that future research should determine whether assortative mating can help reinforce population‐specific selection pressures on coloration. However, to truly test these ideas in our study system, we would need to expand our number of study sites and sample localities closer to the E‐Pirio and D‐Rouvière populations. We would also need to conduct mate choice experiments.

### 4.5 On the processes leading to assortative mating

Assortative mating can be the direct outcome of directional or mutual mate choice (Kraaijeveld et al., 2007). But other processes can also lead indirectly to assortative mating. For instance, if pairs remain together across years or if they choose to live in the same microhabitat, they can experience conditions affecting similarly their plumage. Positive assortative mating can also be created by the scale‐of‐choice effect (Rolan‐Alvarez et al., 2015) when several neighboring populations are sampled and pooled for the purposes of analysis. Despite considering four distinct populations, variations in microhabitat within each population may lead to a scale‐of‐choice effect. Analyses of assortative mating at the individual scale are thus needed to determine the processes driving assortative mating.

Determining these processes was beyond the scope of this paper, aimed at quantifying assortative mating at the population scale. In a parallel study, we have tested at the individual scale several processes comprising mate choice, age, habitat choice, or timing of breeding in our four populations. We found that assortative mating seemed directly due to mate choice, with no effect of indirect processes (Fargevieille, 2016). This result suggests that mate choice is indeed an important process leading to assortative mating.

## 5 Conclusions

First, this long‐term study of assortative mating by two ornaments across multiple populations has revealed that spatiotemporal variation cannot be ignored. Doing so carries the strong risk of generating at erroneous conclusions. Second, the within‐study meta‐analysis approach, which allowed us to account for the dramatic spatiotemporal variation we observed, revealed that assortative mating was positive overall and thus might influence ornament evolution in both sexes. Third, assortative mating demonstrated fine‐scale variation, suggesting it could affect local adaptation and population divergence in our study system, topics that should be explored in future research.

## Conflict of Interest

We finally declare no conflict of interest.

## Data Accessibility

Raw data set for colour trait values in each pair is available from the Dryad Digital Repository: http://dx.doi.org/10.5061/dryad.b1q08.
