# Supplementary material for: Assortative mating by colored ornaments in blue tits: space and time matter
Source: Ecol Evol. 2017 Feb 26;7(7):2069–78. doi: 10.1002/ece3.2822 (PMC5383486; doi:10.1002/ece3.2822)

**Electronic Supplementary Material**

**Appendix 1 Assortative mating over the course of the breeding season**

The aim of this additional analysis is to test whether assortative mating change over the course of the breeding season.

Methods and Results

For some years - three/ four years of our ten years study - we collected feathers during the nest construction period (i.e. just before laying started, which was mid-March for the D-Rouvière, D-Muro, and E-Muro populations and mid-April for the E-Pirio population). At this point, the birds had already become territorial. Only mated pairs for which we had color trait information for both periods—nest construction and chick feeding—were included in the smaller-scale within-study meta-analysis. This restriction explains the relative small sample size in each of the study years (Table S1.1)

| **Table S1.1** Sample sizes (n) for the analysis of assortative mating patterns within breeding seasons | | |
| --- | --- | --- |
| Population | Year | n |
| D-Muro | 2011 | 10 |
|  | 2012 | 13 |
|  | 2013 | 16 |
|  | 2014 | 16 |
| E-Muro | 2012 | 5 |
|  | 2013 | 4 |
|  | 2014 | 6 |
| D-Rouvière | 2008 | 11 |
|  | 2009 | 15 |
|  | 2011 | 7 |
| E-Pirio | 2011 | 10 |
|  | 2012 | 4 |
|  | 2013 | 4 |
|  | 2014 | 8 |

After calculating the raw Pearson correlation coefficients, we carried out a within-study meta-analysis. Year was a random effect, and period (nest construction vs. chick feeding), population, and ornament were fixed effects. The interactions between period and ornament and between population and ornament were included.

Results

The best-fit model (based on DIC values) retained period and population (Table S1.2). Assortative mating increased in strength over the course of the breeding season (Fig. S1.1). However, the population-level patterns were the same as in the full analysis. We thus concluded that the assortative mating patterns and population differences observed in latter were reliable.

| **Table S1.2** Models from the meta-analysis examining assortative mating patterns within breeding seasons. The best-fit model (in bold) had the lowest DIC value. Ornament had two levels—blue crown and yellow chest patch—as did period—nest construction and chick feeding. | | |
| --- | --- | --- |
| Fixed effects | Random effect | DIC value |
| **Period+Population** | **Year** | **-332.8** |
| Ornament*Population+Ornament*Period | Year | -315.6 |
| Population+Ornament | Year | -312.2 |
| Population+Ornament*Period | Year | -310.2 |
| Ornament*Population+Period | Year | -307.8 |
| Population | Year | -306.9 |
| Ornament+Population+Period | Year | -302.3 |
| Ornament*Population | Year | -296.5 |
| Period | Year | -296.2 |
| Ornament+Period | Year | -281.2 |
| Ornament*Period | Year | -271.2 |
| Ornament | Year | -265.5 |
| Intercept | Year | -262.6 |


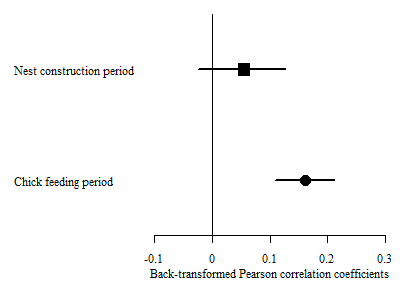
**Figure S1.1** Back transformed Pearson correlation coefficients for the two periods of the breeding season. The values were taken from the model in which period was the sole predictor. The dashed line indicates a coefficient value of zero, or the absence of assortative mating.

**Appendix 2**

| **Table S2.1** Repeatability of color measurements estimated using an ANOVA. n_s_ is the sample size; n_m_ is the total number of measurements (three to six per individual); r_BB_, r_BH_, r_BUVC_, r_YB_, and r_YC_ are the repeatability estimates for blue brightness, blue hue, blue UV-chroma, yellow brightness, and yellow chroma, respectively. The D-Muro and E-Muro populations were pooled into a single category called “Muro.” | | | | | | | | | | |
| --- | --- | --- | --- | --- | --- | --- | --- | --- | --- | --- |
|  |  | Blue crown | | | | | Yellow breast patch | | | |
| Population | Year | n_s_ | n_m_ | r_BB_ | r_BH_ | r_BUVC_ | n_s_ | n_m_ | r_YB_ | r_YC_ |
| Muro | 2005 | 129 | 754 | 0.843 | 0.704 | 0.848 | 130 | 777 | 0.748 | 0.674 |
|  | 2006 | 137 | 815 | 0.801 | 0.785 | 0.845 | 139 | 837 | 0.714 | 0.611 |
|  | 2007 | 0 | 0 | NA | NA | NA | 0 | 0 | NA | NA |
|  | 2008 | 144 | 862 | 0.830 | 0.693 | 0.793 | 148 | 882 | 0.660 | 0.730 |
|  | 2009 | 140 | 828 | 0.843 | 0.802 | 0.884 | 140 | 802 | 0.698 | 0.681 |
|  | 2010 | 188 | 1108 | 0.924 | 0.845 | 0.905 | 188 | 1127 | 0.853 | 0.732 |
|  | 2011 | 287 | 1620 | 0.796 | 0.782 | 0.863 | 280 | 1563 | 0.731 | 0.722 |
|  | 2012 | 301 | 1692 | 0.811 | 0.718 | 0.829 | 308 | 1724 | 0.675 | 0.720 |
|  | 2013 | 254 | 1382 | 0.803 | 0.702 | 0.756 | 269 | 1401 | 0.639 | 0.685 |
|  | 2014 | 312 | 1859 | 0.927 | 0.699 | 0.878 | 312 | 1872 | 0.838 | 0.727 |
| D-Rouvière | 2005 | 224 | 1294 | 0.802 | 0.786 | 0.815 | 166 | 922 | 0.543 | 0.752 |
|  | 2006 | 108 | 601 | 0.681 | 0.717 | 0.807 | 110 | 605 | 0.602 | 0.684 |
|  | 2007 | 186 | 966 | 0.698 | 0.680 | 0.786 | 186 | 1059 | 0.540 | 0.821 |
|  | 2008 | 322 | 1826 | 0.751 | 0.766 | 0.814 | 319 | 1931 | 0.437 | 0.594 |
|  | 2009 | 348 | 1975 | 0.802 | 0.753 | 0.802 | 345 | 1945 | 0.590 | 0.719 |
|  | 2010 | 161 | 952 | 0.918 | 0.742 | 0.871 | 160 | 930 | 0.819 | 0.720 |
|  | 2011 | 209 | 1119 | 0.743 | 0.766 | 0.796 | 203 | 1146 | 0.486 | 0.680 |
|  | 2012 | 154 | 883 | 0.749 | 0.684 | 0.781 | 155 | 852 | 0.504 | 0.612 |
|  | 2013 | 114 | 541 | 0.751 | 0.688 | 0.770 | 104 | 510 | 0.605 | 0.580 |
|  | 2014 | 189 | 1120 | 0.920 | 0.760 | 0.896 | 186 | 1116 | 0.805 | 0.821 |
| E-Pirio | 2005 | 92 | 288 | 0.789 | 0.715 | 0.842 | 99 | 282 | 0.562 | 0.644 |
|  | 2006 | 80 | 434 | 0.729 | 0.681 | 0.777 | 81 | 435 | 0.646 | 0.772 |
|  | 2007 | 128 | 743 | 0.695 | 0.651 | 0.798 | 132 | 739 | 0.509 | 0.687 |
|  | 2008 | 83 | 482 | 0.773 | 0.645 | 0.686 | 81 | 435 | 0.671 | 0.577 |
|  | 2009 | 90 | 523 | 0.854 | 0.683 | 0.735 | 88 | 522 | 0.633 | 0.719 |
|  | 2010 | 110 | 650 | 0.818 | 0.684 | 0.835 | 111 | 666 | 0.772 | 0.611 |
|  | 2011 | 166 | 915 | 0.688 | 0.672 | 0.720 | 163 | 924 | 0.637 | 0.725 |
|  | 2012 | 140 | 803 | 0.797 | 0.699 | 0.805 | 141 | 761 | 0.580 | 0.600 |
|  | 2013 | 120 | 643 | 0.808 | 0.677 | 0.860 | 118 | 558 | 0.791 | 0.707 |
|  | 2014 | 157 | 926 | 0.907 | 0.723 | 0.862 | 157 | 942 | 0.725 | 0.651 |

| **Table S2.2** Pearson correlation coefficients for the five color traits in the four populations (the values for males and females are italicized and unitalicized, respectively; statistical significance: *p<0.05; **p<0.01; p<0.001). | | | | | | |
| --- | --- | --- | --- | --- | --- | --- |
| Population | Color trait | Blue brightness | Blue hue | Blue UV-chroma | Yellow brightness | Yellow chroma |
| D-Muro | Blue brightness |  | -0.135** | 0.026 | 0.042 | 0.096 |
|  | Blue hue | *0.075* |  | -0.555*** | -0.230*** | -0.037 |
|  | Blue UV-chroma | *-0.219**** | *-0.380**** |  | 0.214*** | 0.212*** |
|  | Yellow brightness | *-0.020* | *-0.103** | *0.138*** |  | -0.239*** |
|  | Yellow chroma | *0.116** | *0.026* | *0.320**** | *-0.253**** |  |
| E-Muro | Blue brightness |  | -0.057 | 0.170** | -0.059 | 0.154* |
|  | Blue hue | *0.057* |  | -0.735*** | 0.061 | -0.148* |
|  | Blue UV-chroma | *-0.027* | *-0.668**** |  | -0.075 | 0.178 |
|  | Yellow brightness | *-0.0002* | *0.161** | *-0.065* |  | -0.293*** |
|  | Yellow chroma | *0.138** | *-0.077* | *0.105* | *-0.386**** |  |
| D-Rouvière | Blue brightness |  | -0.005 | -0.089* | 0.102* | 0.263*** |
|  | Blue hue | *0.218**** |  | -0.523*** | -0.120** | 0.084 |
|  | Blue UV-chroma | *-0.384**** | *-0.463**** |  | -0.107* | -0.083 |
|  | Yellow brightness | *0.0987** | *-0.126*** | *-0.044* |  | -0.123** |
|  | Yellow chroma | *0.217**** | *0.091** | *-0.056* | -0.*018* |  |
| E-Pirio | Blue brightness |  | -0.101* | 0.064 | 0.183*** | -0.096 |
|  | Blue hue | *0.082* |  | -0.329*** | -0.191*** | 0.093 |
|  | Blue UV-chroma | *-0.131*** | *-0.418**** |  | -0.018 | 0.020 |
|  | Yellow brightness | *0.215**** | *-0.204**** | *-0.063* |  | -0.302*** |
|  | Yellow chroma | *0.006* | *0.156*** | *0.154*** | *-0.426**** |  |

| **Table S2.3.** Coefficients from the commonality analysis performed for each color trait. Using additive multiple regression models, we tested the ability of each male color trait to predict the values of each female color trait .The process was then repeated using the female color traits as the predictor variables. “Unique” refers to the variance explained uniquely by the trait tested, while “common” refers to the variance jointly explained by the association of ornament-specific traits (e.g., the ability of male blue brightness, male blue hue, and male blue UV-chroma together to account for variance in female blue brightness); r² and r²_adj_ are the two estimates of the proportion of the variance explained by the full model. The highest value for each model is in bold. | | | | | | |
| --- | --- | --- | --- | --- | --- | --- |
| **a**/ |  | Female blue brightness | Female blue hue | Female blue UV-chroma | Female yellow brightness | Female yellow chroma |
|  | r² | 0.123 | 0.166 | 0.274 | 0.113 | 0.236 |
|  | r²_adj_ | 0.120 | 0.163 | 0.271 | 0.110 | 0.234 |
| Male blue brightness | Unique | **0.0924** | 0.0078 | 0.0207 | 0.0063 | 0.0008 |
|  | Common | 0.0013 | -0.0013 | -0.0181 | 0.0048 | 0.0049 |
| Male blue hue | Unique | 0.0046 | **0.1398** | 0.0094 | 0.0059 | 0.0016 |
|  | Common | 0.0019 | -0.0046 | 0.0084 | 0.0028 | -0.0005 |
| Male blue UV-chroma | Unique | 0.0093 | 0.0093 | **0.2098** | 0.0002 | 0.0005 |
|  | Common | -0.0017 | -0.0018 | 0.0158 | -0.0001 | 0.0045 |
| Male yellow brightness | Unique | 0.0129 | 0.0031 | 0 | **0.087** | 0.0021 |
|  | Common | 0.009 | 0.0027 | 0.0007 | 0.0121 | 0.0032 |
| Male yellow chroma | Unique | 0.0002 | 0.0072 | 0.0101 | 0.0007 | **0.216** |
|  | Common | 0.0003 | -0.0035 | 0.0328 | 0.0014 | 0.015 |
| **b**/ |  | Male blue brightness | Male blue hue | Male blue UV-chroma | Male yellow brightness | Male yellow chroma |
|  | r² | 0.107 | 0.148 | 0.264 | 0.111 | 0.259 |
|  | r²_adj_ | 0.104 | 0.145 | 0.262 | 0.108 | 0.257 |
| Female blue brightness | Unique | **0.0821** | 0.0022 | 0.0015 | 0.0112 | 0.0004 |
|  | Common | 0.0121 | 0.0041 | -0.0004 | 0.0077 | 0.0003 |
| Female blue hue | Unique | 0.001 | **0.1146** | 0.0377 | 0.0032 | 0.0033 |
|  | Common | 0.0054 | 0.0228 | -0.0302 | 0.0029 | 0.0003 |
| Female blue UV-chroma | Unique | 0.0003 | 0.0044 | **0.2522** | 0.0025 | 0.0261 |
|  | Common | 0.0021 | 0.0135 | -00.268 | -0.0019 | 0.0168 |
| Female yellow brightness | Unique | 0.0068 | 0.0023 | 0.0016 | **0.076** | 0.0056 |
|  | Common | 0.0047 | 0.0072 | -0.0014 | 0.0193 | -0.0042 |
| Female yellow chroma | Unique | 0.0034 | 0.0006 | 0.0003 | 0.0002 | **0.2113** |
|  | Common | 0.0016 | 0.0002 | 0.0042 | 0.0049 | 0.0153 |


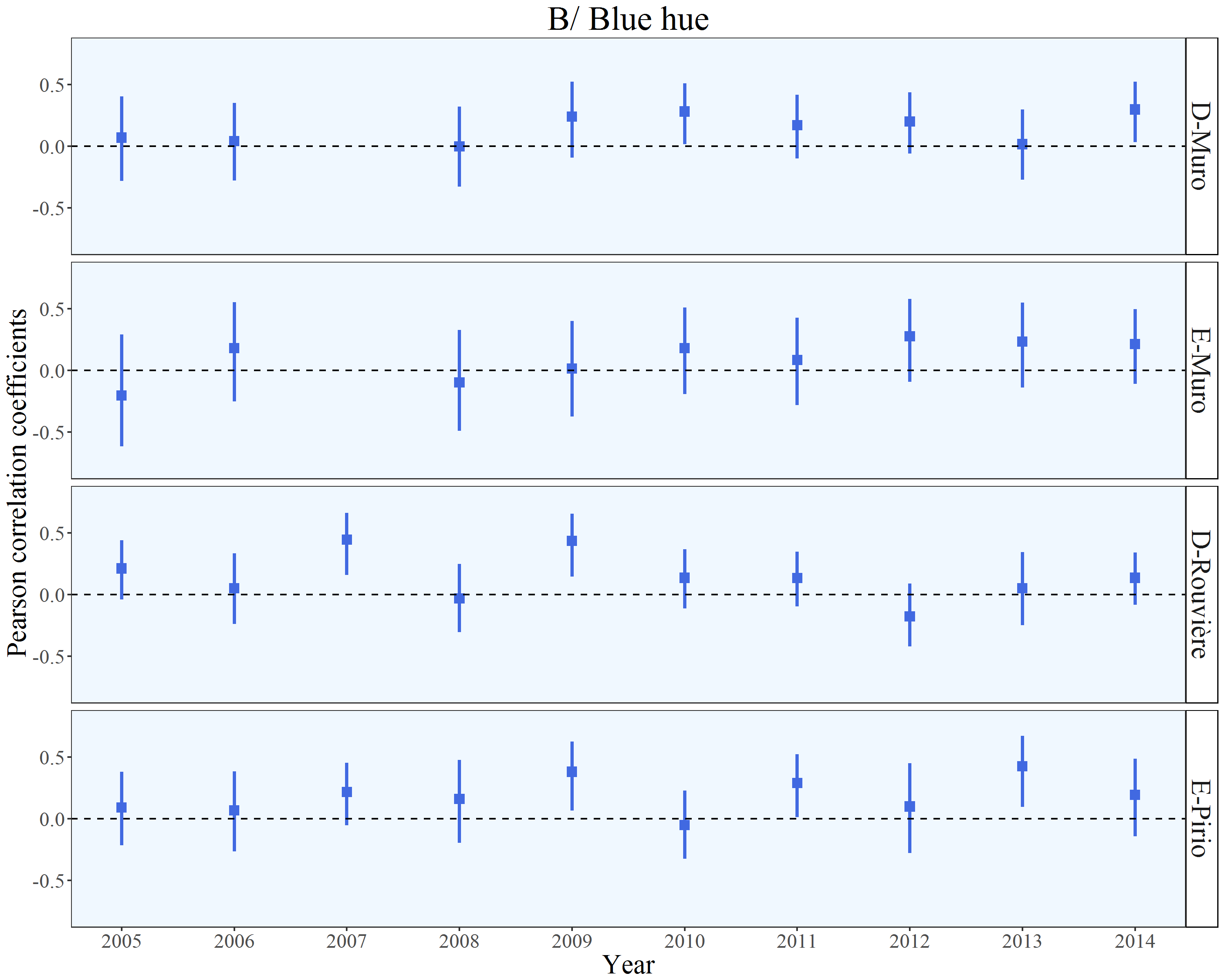

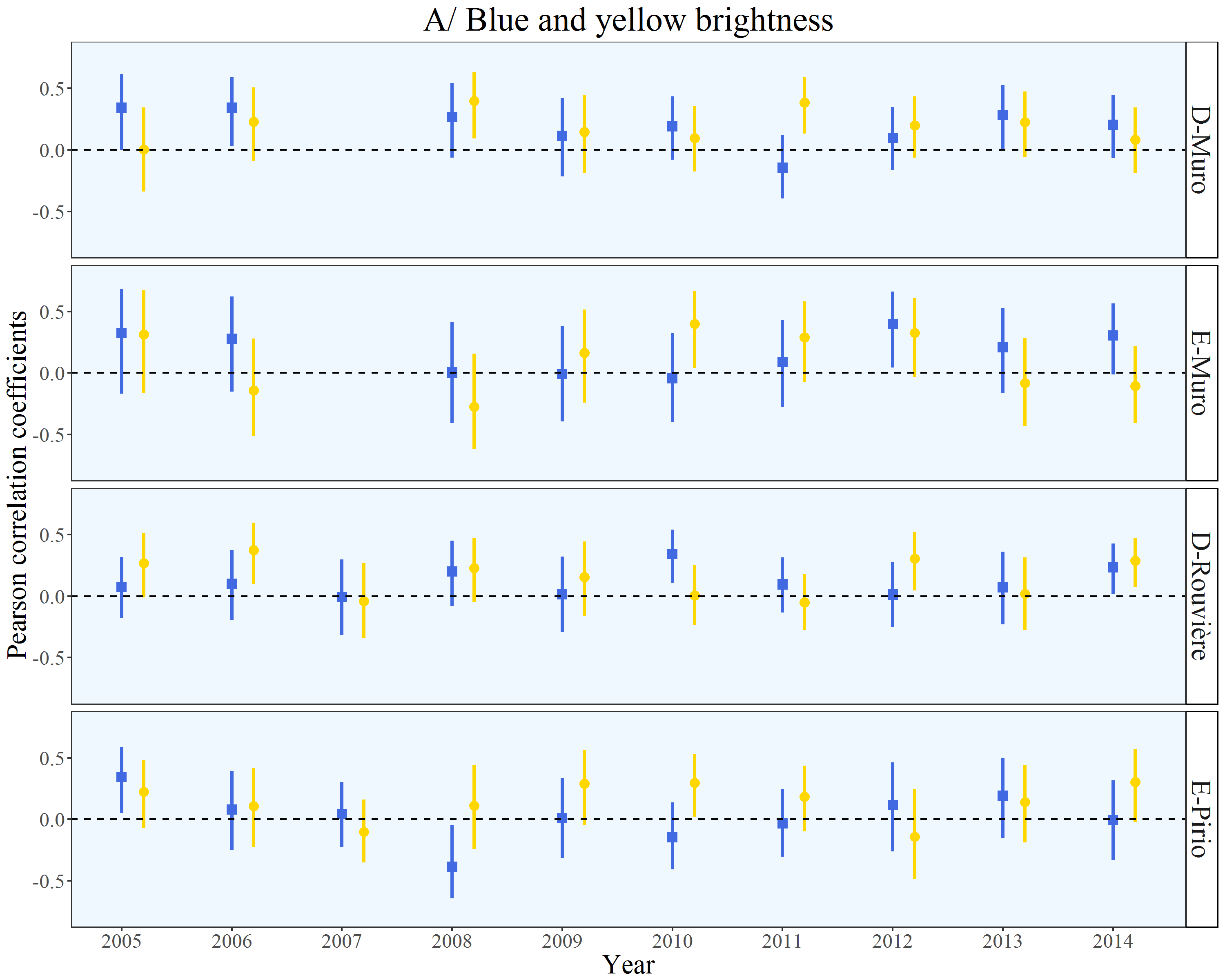
**Figure S2.1** Pearson correlation coefficients for each population for each year for A/ blue brightness (blue squares) and yellow brightness (yellow circles) and B/ blue hue. The bars are the 95% confidence intervals. The dashed lines indicate a coefficient value of zero, or the absence of assortative mating.

| **Table S2.4** Meta-analysis models, in which year was a fixed effect. The best-fit model (in bold) was the model with the lowest DIC value. Ornament had two levels: blue crown and yellow chest patch. | |
| --- | --- |
| Fixed effect | DIC value |
| **Ornament+Year+Population** | **-456.8** |
| Year+Population | -453.9 |
| Population | -451.8 |
| Population+Ornament | -449.0 |
| Year*Population | -443.6 |
| Ornament | -442.5 |
| Ornament*Year+Population | -437.7 |
| Ornament+Year | -437.4 |
| Ornament*Year+Population*Year | -435.2 |
| Ornament*Year | -434.5 |
| Intercept | -433.8 |
| Year | -431.5 |
| Ornament+Population*Year | -426.7 |

**Figure S2.2** Back transformed Pearson correlation coefficients revealing the additive effects of population and year. The different study populations are represented with different symbols and different colors: D-Muro (squares, light green), E-Muro (solid circles, olive green), D-Rouvière (diamonds, dark green), and E-Pirio (open circles, black). The bars are the 95% confidence intervals. The dashed line indicates a coefficient value of zero, or the absence of assortative mating.


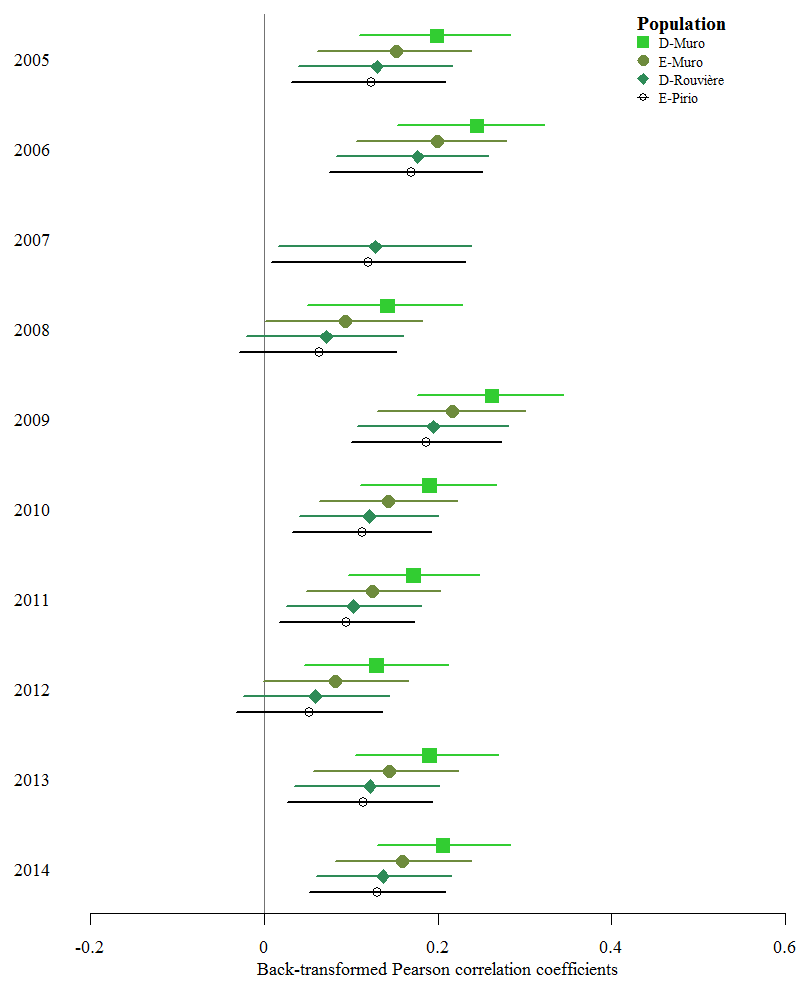

Supplement: Supplementary file 1 [file ECE3-7-2069-s001.docx]
